# The Role of Arginine-Vasopressin in Stroke and the Potential Use of Arginine-Vasopressin Type 1 Receptor Antagonists in Stroke Therapy: A Narrative Review

**DOI:** 10.3390/ijms24032119

**Published:** 2023-01-20

**Authors:** Karol Chojnowski, Mikołaj Opiełka, Jacek Gozdalski, Jakub Radziwon, Aleksandra Dańczyszyn, Andrew Vieira Aitken, Vinicia Campana Biancardi, Paweł Jan Winklewski

**Affiliations:** 1 Student Scientific Circle of the Department of Adult Neurology, Medical University of Gdansk, 17 Smoluchowskiego Street, 80-214 Gdansk, Poland; 2 Department of Adult Neurology, Medical University of Gdansk, 17 Smoluchowskiego Street, 80-214 Gdansk, Poland; 3 Department of Anatomy, Physiology, and Pharmacology, College of Veterinary Medicine, Auburn University, Auburn, AL 36849, USA; 4 Center for Neurosciences Initiative, Auburn University, Auburn, AL 36849, USA; 5 Department of Human Physiology, Medical University of Gdansk, 15 Tuwima Street, 80-210 Gdansk, Poland; 6 2nd Department of Radiology, Medical University of Gdansk, 17 Smoluchowskiego Street, 80-214 Gdansk, Poland

**Keywords:** arginine-vasopressin, vasopressin receptors, copeptin, ischemic stroke, stroke pathophysiology, acute stress response, neuroendocrine dysfunction, cerebral edema, neuroinflammation, blood-brain barrier

## Abstract

Stroke is a life-threatening condition in which accurate diagnoses and timely treatment are critical for successful neurological recovery. The current acute treatment strategies, particularly non-invasive interventions, are limited, thus urging the need for novel therapeutical targets. Arginine vasopressin (AVP) receptor antagonists are emerging as potential targets to treat edema formation and subsequent elevation in intracranial pressure, both significant causes of mortality in acute stroke. Here, we summarize the current knowledge on the mechanisms leading to AVP hyperexcretion in acute stroke and the subsequent secondary neuropathological responses. Furthermore, we discuss the work supporting the predictive value of measuring copeptin, a surrogate marker of AVP in stroke patients, followed by a review of the experimental evidence suggesting AVP receptor antagonists in stroke therapy. As we highlight throughout the narrative, critical gaps in the literature exist and indicate the need for further research to understand better AVP mechanisms in stroke. Likewise, there are advantages and limitations in using copeptin as a prognostic tool, and the translation of findings from experimental animal models to clinical settings has its challenges. Still, monitoring AVP levels and using AVP receptor antagonists as an add-on therapeutic intervention are potential promises in clinical applications to alleviate stroke neurological consequences.

## 1. Introduction

Stroke results from an acute central nervous system injury caused by the disruption of cerebral blood flow or bleeding within or around the brain, with consequent neurological damage and loss of function. Ischemic stroke due to cerebral blood vessel occlusion comprises most of the cases (~62% of all new strokes in 2019), followed by intracerebral hemorrhage stroke (28%), and subarachnoid hemorrhage stroke (10%) [1]. Independent of the cause, in 2019 stroke remained the second-leading cause of death globally and the third-leading cause of disability and all-death mortality combined [1]. 

The extent of the neurological injury in response to stroke, particularly ischemic stroke as the most common type, depends on various factors, including the severity, duration, and area affected. The pathological mechanisms are complex and the time window to intervene at each stage is limited. With the decrease of cerebral blood flow, oxygen and glucose deprivation that follow change the ionic environment, thus promoting excitotoxicity and neuronal loss [2]. The resulting blood-brain barrier (BBB) disruption, increased levels of reactive species, and neuroinflammation all contribute to poor outcomes in ischemic stroke. In the post-stroke phase, a chronic neuroinflammatory process might occur at the infarct core and distal regions, promoting long-term neuronal tissue damage. A subsequent stroke-induced secondary neurodegeneration may occur in the distal regions. Neurodegeneration may lead to loss or impairment of motor function, autonomic and/or cognitive systems depending on the functional location of neuronal loss and the structure affected [3]. 

Despite scientific advances in understanding the mechanisms of stroke, etiology, and prognosis, effective treatments are still deficient. For instance, though mitigating post-stroke neuroinflammation is promising in improving stroke outcomes, multiple neuroprotectants have failed clinical efficacy to date [2]. Hence, a constant need to search for novel or complementary targets to decrease the burden of stroke. Likewise, long-term prognosis-effective biomarkers remain a cause for concern.

In this narrative review, we present several pieces of evidence suggesting arginine vasopressin (AVP) neuropeptide receptors as a potential therapeutic target, and copeptin, a surrogate marker of AVP, as an effective biomarker for long-term prognosis and risk stratification in stroke. 

AVP is a neuropeptide critically involved in the maintenance of body homeostasis. The primary physiological mechanism controlling AVP release to the circulation is osmotic stimuli, followed by nonosmotic stimuli, including blood volume and pressure changes [4]. However, AVP secretion also occurs during stress-related stimuli associated with acute onset diseases, including stroke, in a hypersecretion manner [5,6]. As a neurotransmitter and neuromodulator within the central nervous system (CNS), AVP plays significant roles in maintaining physiological cerebral fluid balance, electrolyte homeostasis, and vascular resistance [5,7,8,9,10]. All those functions are highly affected by a cerebrovascular accident, suggesting that dysregulated AVP release may play a significant role in stroke pathophysiology. Indeed, AVP hypersecretion is implicated in major stroke-related complications, including brain edema, vasoconstriction, oxidative stress, BBB disruption, and neuroinflammation [11,12,13,14]. 

The AVP precursor peptide (pre-proAVP) is synthesized mainly within neurosecretory neurons of the hypothalamus and subsequently transported along the axons to the neurohypophysis. Proteolytic cleavage during axonal transport forms the mature AVP alongside copeptin and dissociates their intracellular carrier protein neurophysin II in equimolar amounts, independent of the stimuli applied [15,16,17]. In contrast to AVP’s short half-life and unstable nature, copeptin is stable in the serum and plasma of patients with elevated AVP, reliably reflecting AVP circulating concentrations [18]. Hence, copeptin has been used as a surrogate marker for AVP in multiple disease states, including myocardial infarction and stroke [18]. Recently, studies exploring the detrimental role of AVP in animal models of ischemic and hemorrhagic stroke have supported a positive correlation between copeptin level and stroke severity. Moreover, an association between increased levels of AVP in stroke patients and poor prognosis has been established [19,20,21,22,23,24,25]. Hence, from a clinical perspective, the importance of understanding further the AVP involvement in stroke-elicited damage progression as copeptin, as it is secreted in equimolar concentrations as AVP and has been considered a surrogate marker of AVP, is an effective biomarker in terms of risk stratification after ischemic stroke and in predicting vascular events after a transient ischemic attack (TIA) [21,26]. 

Another clinical implication in understanding AVP stroke pathology in stroke settings is using AVP receptor antagonists as an add-on or complementary target to mitigate AVP-induced edema and BBB disruption [26,27,28,29,30,31,32,33]. AVP receptor antagonists have been FDA-approved for hyponatremia, and hence are readily available for repurposing [34,35]. 

In this narrative review, we first briefly summarize the physiological role of AVP. We then discuss the underlying mechanisms contributing to AVP hypersecretion during the onset of the cerebrovascular accident and how this phenomenon causes detrimental effects on both systemic and local scales. Furthermore, recent data suggesting a promising role for copeptin use as a prognostic tool in stroke is introduced. Finally, we summarize current evidence for using selective and mixed AVP receptor antagonists in the acute phase of ischemic stroke and as an add-on therapy for mitigating post-stroke complications. The discussion is primarily focused on AVP in ischemic stroke, as most data has studied this type of stroke. Our goal is to provide the reader with a comprehensive, state-of-the-art narrative review highlighting AVP’s critical role in stroke pathology and its potential for therapeutic targets.

## 2. The Physiological Role of Arginine Vasopressin

AVP is synthesized in magnocellular neurons located in the supraoptic and paraventricular nucleus of the hypothalamus (PVN). The AVP gene encodes the precursor protein pre-proAVP, which is proteolytically cleaved during axonal transport towards the pituitary gland to form AVP and copeptin in equal concentrations [15,16]. Physiological secretion of AVP to the circulation occurs primarily in response to osmotic stimuli, sensed mainly by osmoreceptors and through nonosmotic cues detected by baroreceptors and atrial stretch receptors [4]. Once released, circulating AVP acts as a hormone in widespread targets regulating many physiological functions to promote fluid homeostasis by controlling water balance and plasma ion concentration, and maintaining systemic blood pressure, by controlling the vascular tonus [4].

AVP acts primarily through AVP activation of G protein-coupled receptors, namely: V1a (V1aR) and V2 receptors (V2R), to promote its physiological functions [8]. AVP acting on V2R found in the distal tubules and collecting ducts of the kidney regulates proper fluid exchange and plasma osmolality within the renal system [36]. The AVP/V2R activation causes upregulation of aquaporin 2 (AQP2), resulting in ion gradient-driven water influx and subsequent urine concentration, plasma dilution, and plasma osmolality reduction [36]. 

Blood pressure regulation by AVP is complex and still not fully understood, given that AVP has interactions with other systems, such as the sympathetic and the renin-angiotensin system, both critical regulators of blood pressure. Still, AVP predominantly regulates blood pressure through V1aR and V2R, located on endothelial and vascular smooth muscles. Their activation by AVP causes either vasoconstriction or vasodilation, depending on the vascular bed [37], and aids blood pressure maintenance in normotensive and hypertensive subjects [38,39]. Moreover, AVP vasoconstriction response depends upon arteriolar diameters. As such, the vasoconstrictive response to AVP in large arterioles is more significant than that observed by norepinephrine treatment [40].

On the other hand, smaller arterioles do not show differences between treatments in rodents’ microcirculation [40]. Hence, AVP has clinical importance and use in advanced hypovolemic or vasodilatory shock states [41].

In the CNS, AVP acts as a neurotransmitter and neuromodulator and participates in various physiological and behavioral functions, as summarized in the remainder of this section. In concert with the corticotropin-releasing hormone, AVP regulates corticosteroids’ secretion within the CNS and periphery, contributing to the endocrine stress response [42,43]. In the CNS, AVP regulates the hypothalamic-pituitary-adrenal (HPA) axis through a third characterized receptor named V1b located in the corticotropic cells in the anterior pituitary gland (V1bR, also called V3) [44]. Additionally, AVP directly activates V1aR in the adrenocortical cells to release adrenocorticotropic hormone (ACTH), hence potentiating the release of cortisol, an essential hormone involved in stress response [45,46].

Moreover, AVP is present within the so-called intrinsic vasopressin fiber system composed of astrocytes and astrocyte-like cells. Although the precise function thereof remains unknown, it is postulated that the vasopressin fiber system facilitates neocortical water flux via AQP4 in response to V1aR activation. Thus, playing a role in brain water regulation and ion homeostasis [26,47]. 

In the medial preoptic area (MPO), a hypothalamic nucleus containing AVP-activated (AVP+) neurons, AVP stimulates γ-aminobutyric acid (GABA) release. Local, chemogenic stimulation of all MPO AVP+ neurons induces local hyperthermia while decreasing endotoxin or stress-activated fever with a generally antipyretic effect [48,49,50].

Within the suprachiasmatic nuclei (SCN), daily rhythm regulation is tightly connected to AVP signaling and HPA activity [51]. HPA-regulated cortisol increase might be inhibited when the AVP is derived from parvocellular SCN neurons or stimulated if derived from parvocellular PVN neurons [52,53]. Although AVP’s role in daily rhythm regulation has not been clearly defined, based on recent bioluminescence findings, AVP signaling influences SCN period, precision, and organization [54]. 

AVP affects neuronal oscillations generated at various scales, from individual neurons through local networks to multiple neural systems across the brain [55]. Among other functions, AVP activation translates into fine-tuning neural responses behind emotional reactivity, risk acceptance, cognitive functioning, social interactions, and maternal behavior in mammals besides oxytocin [56,57]. 

Additionally, AVP is one of the modulators of neurogenesis. In rat models, brain AVP content gradually increases from 16 days of pregnancy, while pituitary AVP rapidly increases from 19 days until birth [58]. Trials on rodents showed that lower AVP concentration neonatally is associated with reduced brain weight, mainly affecting the cerebellum. In 10–15% of cases, these abnormalities persist throughout life [58].

## 3. Arginine Vasopressin Hypersecretion and Release Mechanisms during Stroke

Several studies indicate excess release of AVP in association with somatic and psychological stressors present in acute onset diseases, including myocardial infarction, sepsis, and stroke [59]. Interestingly, the stress-related release of AVP in response to pathological stimuli shows a much higher increase in magnitude than classical osmotic stimuli. For instance, in the baboon model of hemorrhagic shock, baseline copeptin level (reflecting AVP) increased approximately 36-fold (median level from 7.5 to 269 pmol/L) [60].

Indications of increased release of AVP in ischemic stroke were reported in animal models of stroke and stroke patients. AVP gene and protein expression were reported as elevated within the SON and PVN in an experimental model of cerebral ischemia and reperfusion, suggesting transcription and translation of AVP are increased in acute brain injury [61]. Patients with ischemic stroke show a significant increase of AVP in plasma and cerebrospinal fluid [62,63,64,65]. Elevated AVP levels in blood plasma were observed in patients with ischemic stroke during the 24 h period [63]. The observed AVP hypersecretion was independent of plasma osmolality and mean arterial pressure and correlated with the mean size of the lesion and level of neurological deficit. However, no correlation was observed between AVP release and vascular territory of ischemic injury [63].

During the acute phase of stroke, secretion of AVP occurs mainly from neurosecretory MNCs neurons located in the PVN and SON, as the AVP neurons are resilient to ischemia and are capable of secreting AVP after ischemic insult [66,67]. However, other local sources of AVP during brain injury were also identified, including activated microglia, choroid plexus, and to a lesser extent, brain endothelial cells [62,68]. Notably, AVP release in experimental stroke was found in the infarct and peri-infarct spatial location areas [62,66]. 

In the acute phase of ischemic stroke, overactivation of the angiotensin-converting-enzyme (ACE)/angiotensin (Ang II)/angiotensin receptor 1 (AT1R) axis, sympathetic and baroreceptor dysregulation are frequently observed. Such dysregulation causes blood pressure fluctuations, impairs cerebrovascular autoregulation, increases pro-inflammatory cytokine production in the parenchyma, and induces hyperglycemia [67,69,70,71,72]. The state of sympathetic predominance after stroke can cause catecholamines release, which activates the magnocellular neurons in PVN and SON, resulting in AVP release [73,74]. Moreover, the state of hyperglycemia following stroke (as a result of direct post-stroke hyperglycemia or exacerbation of pre-existing diabetes) may increase the number of AVP-expressing neurons in PVN and SON, resulting in increased release of AVP during stroke [75,76]. Another nonosmotic systemic stimulus contributing to AVP release during stroke is the increased intracranial pressure (ICP) [77]. ICP in stroke was classically attributed to cerebral edema formation after stroke [78]. However, recent evidence demonstrates that increased ICP is also associated with the acute phase of stroke before the edema formation [79]. In stroke, mechanical pressure created before/during edema formation exerts direct pressure on the hypothalamus, which results in AVP release from PVN and SON [77]. 

The local mechanisms contributing to AVP release during stroke include glutamate release, local hyperosmotic environment formation, structural and functional changes in astrocytes located in PVN and SON, and pro-inflammatory mediators’ release. Excessive and prolonged glutamate release occurs during stroke in infarct and peri-infarct areas, leading to exaggerated depolarization of postsynaptic neurons and neuronal death through excitotoxicity [80]. After the stroke, elevated pools of circulating glutamate are detected in patients’ plasma and cerebrospinal fluid, which can directly interact with AVP neurons in PVN and SON [81,82]. AVP neurons in the hypothalamus receive dense glutaminergic innervation, accounting for approximately 25% of the total number of synapses formed in PVN and SON. Previous work shows glutamate induces AVP release in conscious rats by stimulating non-n-methyl-d-aspartate (NMDA) receptors [83]. Furthermore, after middle cerebral artery occlusion (MCAO) in rats, glutamate levels rapidly increase in PVN and SON in the first 15 min after the procedure. 

Moreover, ischemic stroke induces increased BBB permeability, enabling the passage of ions, molecules, and fluids into the brain parenchyma, disrupting ion homeostasis [14]. Particularly after ischemic injury, an increase in BBB Na+/H+ exchangers, Na+-K+-Cl− cotransporters, or the calcium-activated potassium channel KCa3.1, induces the uptake of the Na+ ions in the brain parenchyma [14,84]. Consequently, the formation of a local hyperosmotic environment in PVN and SON can enhance the release of AVP [85]. 

The release of AVP during stroke is also regulated by interactions of astrocytes and microglia with magnocellular neurons located in PVN and SON. In physiological conditions, the activity of AVP neurons is under negative feedback modulation from astrocytes [86,87]. Increased AVP secretion in PVN and SON increases the aquaporin 4 (AQP4) expression, which is associated with the extension of glial fibrillary acidic protein (GFAP) filaments. Therefore, astrocytic processes can reversely expand around AVP neurons and inhibit AVP secretion [87]. However, during stroke, the maladaptation of astrocyte plasticity causes the regulatory volume decrease of astrocytes, followed by the release of glutamate into the extracellular space [88]. It has been demonstrated that MCAO and basilar artery occlusion (BAO) reduce the GFAP and AQP4 in astrocytes located around AVP neurons, followed by the separation of GFAP from AQP4, thereby increasing the activity of AVP neurons [89,90].

Additionally, activated microglia and astrocytes can release many pro-inflammatory mediators during stroke [91]. Previous works indicate that AVP can be released during a cerebral injury in response to TNFa, IL-1, and IL-6. Furthermore, AVP acts synergistically with those cytokines and increases the local release of CXC and CC chemokines [92].

Following the hypersecretion of AVP during stroke onset, an early (1–2 h) increase of V1aR is observed in astrocytes, neurons during axonal beading, and brain endothelium [6,31,93]. An increase in V1aR expression is correlated with AQP4 upregulation. The V1aR is also redistributed from the astrocyte body to the astrocytic processes, where the AQP4s are localized [90]. Consequently, the injured brain parenchyma is more susceptible to AVP-induced effects, particularly brain edema formation. 

## 4. Arginine Vasopressin Contribution to Ischemic Stroke Pathomechanism

### 4.1. Systemic Effects of AVP in Stroke

#### 4.1.1. Arginine Vasopressin and Hyponatremia 

The detrimental effects of AVP overproduction can be observed in patients with the syndrome of inappropriate antidiuretic hormone secretion (SIADH), which is commonly associated with stroke (3.9–45.3% and 40–45% on admission and during hospitalization, respectively) [94,95]. AVP’s action to effectively lower the plasma osmolality is mainly focused on reducing the concentration of sodium, a predominant osmolyte. Thus, a pathological increase in AVP secretion results in hyponatremia. Of note, although AVP induces water conservation in the kidneys, the regulatory mechanisms, i.e., the renin-angiotensin system, prevent extracellular volume expansion. Therefore, the elevated levels of AVP observed in SIADH result in the pathological state of euvolemic hyponatremia [96]. 

The severity of CNS damage caused by hyponatremia is causally associated with the magnitude of sodium deficiency and the rate of change in plasma sodium concentration [96]. In severe cases of hyponatremia, the hypoosmotic state produces a gradient-driven water influx into the brain causing cerebral edema [97]. The edema and increased intracranial pressure (ICP) promote further AVP secretion followed by edema accretion [26,77]. For instance, posterior reversible encephalopathy syndrome (PRES), a clinicoradiological condition characterized by brain edema as a primary symptom was associated with an increased AVP secretion [98]. 

As mentioned above, nearly half of stroke patients develop hyponatremia either on admission or during hospitalization [94]. Moreover, sodium concentration below 135 mmol/L is associated with higher mortality and larger baseline intracerebral hemorrhage volume [99,100,101,102]. Although there are many overlapping causes of low sodium levels in stroke patients, AVP’s role seems significant, especially in hemorrhagic stroke. Indeed, in ischemic stroke and mild/moderate subarachnoid hemorrhage cases, 7% and 71% of hyponatremia cases were attributed to SIADH, respectively [103,104].

#### 4.1.2. Arginine Vasopressin, Autonomic Nervous System, Inflammation, and Immunosuppression Response

Strokes may provoke damage to anatomical structures of the autonomic nervous system, causing autonomic dysregulation. Likewise, AVP acts centrally, coordinating autonomic responses, i.e., AVP administered intraventricularly in rodent models causes sympathetic activation [105,106,107]. AVP-mediated sympathetic activation and stroke-elicited disruption of central control of the autonomic nervous system could account for catecholamine release, and thus immunosuppression. The diminished immune response can be detrimental in stroke patients due to the increased risk of infection complications and potential bystander autoimmune response [108]. 

AVP is secreted in response to IL-6, an inflammatory cytokine released during infection. This temporal relationship may explain the hyponatremia associated with high CRP values [109]. Moreover, AVP modulates immune response by increasing IFN-y and primary antibody production in inflammatory cells. Furthermore, AVP stimulates the release of prolactin, a hormone with pro-inflammatory properties [110]. This data suggests the involvement of AVP in inflammatory response exacerbation (caused by, e.g., hospital-acquired infection), which may be related to mid-term stroke outcome worsening. 

#### 4.1.3. Arginine Vasopressin and Stress Response

The release of AVP, and other stroke-related events, such as cytokine release and ischemic disruption of HPA inhibitory areas, can entail the HPA axis activation [111,112]. Indeed, one of the earliest events following a brain injury caused by ischemia or hemorrhage (depending on the type of stroke) is the activation of the HPA axis and subsequent hypercortisolism [57,112]. As previously mentioned, AVP, in concert with corticotrophin-releasing hormone (CRH), can stimulate the pituitary release of ACTH, subsequently activating adrenal glucocorticoid secretion [45,46]. AVP, in physiological conditions, is a weaker ACTH secretagogue compared to CRH. However, AVP acts synergistically with CRH and causes extensive ACTH release after stimulation by various acute stressors, including stroke [113].

The resulting increase in cortisol starts promptly after the stroke onset and persists from seven days to months after an insult [114]. The effect of cortisol on brain tissue is detrimental and is associated with structural changes in all brain regions, but predominantly gray matter [115]. Mounting evidence suggests that chronic cortisol elevation causes atrophic changes in the hippocampus, leading to hippocampus-dependent learning and memory impairment [116]. The loss of hippocampal volume may be attributable to brain-derived trophic factor expression changes in the hippocampus [117]. Moreover, the prolonged high cortisol levels are associated with an exacerbation of Alzheimer’s disease, which is putatively a result of an increase in oxidative stress and amyloid beta peptide toxicity [118].

Regarding stroke, in the most recent systematic review involving a total of 1340 patients, high cortisol levels at admission were associated with greater dependency, morbidity, and mortality in patients with an ischemic stroke. Nevertheless, there is no evidence of cortisol’s causal role in worsening stroke outcomes to date [114]. Moreover, the degree of involvement of AVP in ischemia-elicited cortisol rise has not yet been clarified.

Hypersecretion of AVP and consequent increase in plasma AVP concentration may lead to depression, including endogenous or major depressive disorder and anxiety due to HPA disorder and following hypercortisolemia [57,112]. The exact biochemical mechanism is not well known; however, there seems to be functional interaction between AVP (via V1aR) and serotonin (via 5-HT receptor) at the level of the hypothalamus. This association might be significant in aggressive behavior as well [119,120]. Such behavior often exists in stroke patients during the acute phase, who may experience depressive mood, aggression, or hostility even if they have never been diagnosed with depression or other mood disorders before [121]. 

#### 4.1.4. Arginine Vasopressin and Platelet Aggregation

AVP-induced platelet aggregation is facilitated by V1R activation and subsequent thromboxane release [122]. However, the effect of AVP on platelets could only be observed in physiologically unattainable concentrations of AVP. Moreover, there is no link between AVP and platelet activation in human pathology [123]. Thus, the significant influence of AVP on platelet aggregation in stroke is theoretically unlikely and unsubstantiated.

### 4.2. Central Nervous System Local Effects of Arginine Vasopressin System in Stroke

Hydromineral disturbance and neurovascular unit damage are strongly associated with stroke. Moreover, phenomena such as BBB disruption, neuroinflammation, astrocyte, and neuronal swelling, take place during the acute phase of the ischemic stroke and account for brain edema formation (Table 1) [11]. Under the term “brain edema” associated with ischemic/hemorrhagic stroke, cytotoxic and vasogenic edema can be distinguished [11,124]. Although both forms of edema coexist simultaneously, the former dominates in ischemic stroke, whereas the latter is associated with hemorrhagic stroke [125]. The water exchange between vascular and perivascular space and between perivascular and cellular space is regulated by AQP4, a water channel expressed mainly on astrocytes and endothelial cells [126]. In cytotoxic edema, AQP4 upregulation causes water movement from extracellular space into the astrocytes aggravating astrocyte compensatory swelling, thus increasing brain edema [127,128,129]. However, in vasogenic edema, AQP4 upregulation aids water movement from brain interstitial fluid to the perivascular drainage system, i.e., the glymphatic system, thus attenuating the damaging effects of local edema [125,130,131,132]. The data on AVP’s influence on AQP4 expression is unclear as studies show its up-regulatory and down-regulatory effects [31,33,133]. 

Regarding ischemic stroke, in one study, treatment with a V1R antagonist increased AQP4 expression [134]. Contrarily, in a study on intracranial hemorrhage animal models, V1aR antagonists led to the downregulation of the AQP4 expression [135]. Interestingly, in both of these studies, AVP receptor antagonism proved beneficial in brain edema attenuation [134,135]. It is clear that further studies are needed to address differences between ischemic and hemorrhagic stroke regarding AVP-mediated AQP4 expression modulation and edema formation.

AVP influences ion balance in astrocytes and endothelial cells. The ion imbalance caused by Na/K pump dysfunction is aggravated by V1aR-mediated luminal ion transporters’ activity enhancement [26,136]. AVP activates endothelial V1R, which results in AMPK and ERK1/2 pathways activation leading to NKCC1, NHE 1, and NHE2 upregulation [26,136,137]. Moreover, AVP enhances sympathetic response, which also stimulates NKCC1 [138]. The resulting influx of sodium into the endothelial cells and astrocytes promotes osmosis-driven water movement into the extracellular space and astrocytes [139]. 

Stimulation of V1aRs found on cerebral vasculature leads to endothelin-1 overexpression and protein kinase C (PKC) pathway activation, which translates into oxidative stress exacerbation and BBB disruption. A study conducted by Faraco et al. has shown that water deprivation-induced AVP hypersecretion elicits AVP-mediated oxidative stress leading to cerebrovascular dysregulation in murine brains [140]. 

Moreover, AVP may damage BBB by stimulating matrix metallopeptidase 9 (MMP9) expression in the brain endothelium [92]. The activation of MMP9 causes proteolytic degradation of the basal lamina, increasing the vessel permeability [141,142]. Furthermore, MMP9 alters the expression of tight junction components, resulting in increased neutrophil and macrophage infiltration through BBB into the brain [143]. The influx of inflammatory cells is augmented by AVP-mediated neutrophil (CXCL1 and CXCL2) and monocyte (CCL2) chemoattractant production in the brain endothelium and astrocytes [92]. AVP also enhances the production of VEGF, a potent vascular permeability-increasing agent, in mesangial cells acting through V1aR, further enhancing BBB leakage [144,145]. 

AVP elicits the vasoconstriction of cerebral blood vessels via V1aR, causing an increase in cerebral perfusion pressure (CPP) [146]. In the setting of SAH-induced AVP release, vasoconstriction causes a decrease in cerebral perfusion [65]. Contrarily, the increase in CPP caused by AVP administration was associated with the improvement of cerebral blood flow in TBI patients [146]. Most probably, the elevated CPP counteracted the dysfunction of cerebral blood flow autoregulation elicited by sympathetic activation and arterioles distention [147,148]. These results support the rationale for further research regarding the influence of AVP on cerebral blood flow in other diseases associated with cerebral autoregulation impairment, including ischemic stroke [149].

In the rodent TBI model, AVP aggravated injury-elicited inflammatory response. Mechanistically, AVP amplifies CXC and CC chemokines synthesis in the endothelium and astrocytes. In line with this, AVP stimulates the expression of high-mobility group box1, a well-known inflammatory cytokine, in astrocytes during hypoxia/reoxygenation [150,151]. A recent study has shown AVP involvement in IL-6 production in murine hearts, which may play a role in myocardial inflammation in heart failure [152]. Taken together, local immunogenic effects of AVP in combination with enhanced immune cell infiltration caused by AVP-mediated BBB disruption and pro-inflammatory mediators’ production may play a significant role in stroke-associated neuroinflammation.

As highlighted in Table 1, several neuropathological mechanisms associated with stroke pathology may benefit from the AVP receptor antagonism as a target to improve secondary mechanisms of stroke.

**Table 1:** Receptor-mediated effects of arginine vasopressin related to stroke pathophysiology.

Mechanism	Involved Receptors	Receptor Location in the Brain	Effects	Authors
Brain Edema Formation	V1aR	Neurons, Glia	▪ Disruption of the hydromineral balance ▪ Redistribution of V1 receptors in astrocytes ▪ AQP-4 channel regulation—increase in brain water retention ▪ Modulation of Na^+^/K^+^ ion channels ▪ Development of Intracranial Hypertension	[6,33,133,153,154,155,156]
Time-dependent Arteries and arterioles vasodilation and vasoconstriction.	V1aR	Smooth muscle layer of brain vasculature; Endothelium	▪ Reduced cerebral blood flow. Increase of CPP, but not CBF ▪ Aggravation of cerebral ischemia.	[145,146,157,158,159,160]
Increased BBB permeability	V1aR	Astrocytes, podocytes, brain endothelium	▪ AVP-mediated stimulation of cerebral microvascular endothelial cell NKCC and NHE ▪ Astrocytic endfeet swelling	[144,145,156,161,162]
AVP potentiating action on the CRH-evoked ACTH secretion.	V1bR (V3)	Adenohypophysis	▪ Cortisol release, hypercortisolism	[163,164]
Post-stroke, edema-associated inflammation	V1aR	Astrocytes	▪ Stimulation of excessive AVP release by inflammatory cytokines including IL-6 ▪ Hmgb1, Prx2, and Tlr2 upregulation by AVP ▪ Synergistic AVP action with TNF-alpha on chemokines release	[92,150,165]

AVP, arginine vasopressin; V1aR, Vasopressin receptor 1A; V1bR (V3), Vasopressin receptor 1b (V3 pituitary receptor); AQP-4, Aquaporin-4; CPP, Cerebral perfusion pressure; CBF, Cerebral blood flow; BBB, Blood-brain barrier; CRH, Corticotropin-releasing hormone; ACTH, Adrenocorticotropic hormone; NKCC, Na-K-Cl cotransporter; NHE, Na/H exchanger; IL-6, Interleukin- 6; Hmgb1, High mobility group box 1 protein; Prx2, peroxiredoxin 2; Tlr2, Toll-like receptor-2; TNF-alpha, Tumor necrosis factor alpha.

## 5. The Prognostic Value of Copeptin in Acute Stroke Patients 

As summarized above, increases in AVP are detrimental to stroke and have a considerable potential of being used as a therapeutic target as the mechanisms are directly associated with AVP receptors. Furthermore, although AVP is an unstable peptide, it is released in equimolar concentrations with copeptin and validated as a surrogate marker of AVP.

Clinically, copeptin measurements in acute ischemic stroke serve as a tool to predict functional outcomes [20,166]. A meta-analysis of the correlation between copeptin levels and functional outcomes in acute ischemic stroke has shown that a 10-fold increase in copeptin level was associated with a higher risk of poor 3-month and 1-year outcomes (OR = 2.56, 95% CI: 1.97–3.32) and all-cause mortality (OR = 4.16, 95% CI: 2.77–6.25) [24]. Moreover, the authors observed that using copeptin levels in addition to the National Institutes of Health Stroke Scale (NIHSS) score provided a better prediction for unfavorable outcomes than using the NIHSS score alone, showing the importance of such predictive biomarkers in everyday clinical practice [24]. Accordingly, De Marchis and colleagues recently proposed a scale for risk stratification based on clinical features and copeptin levels [167]. The CoRisk score, available for calculation online, considers the patient’s age, NIHSS score, copeptin plasma concentration levels, and information on whether the patient received intravenous therapy [168]. A validation study of the CoRisk score scale showed good sensitivity in outcome prediction, with three in four patients classified correctly, with an area under the curve (AUC) of 0.819 [167]. 

Another promising application of copeptin measurement is to predict stroke after an episode of TIA. Previous studies have shown that patients with a higher copeptin level measured immediately after the TIA event are at a greater risk of developing a stroke or any cerebrovascular re-event [21,169]. In addition, in a long-term follow-up of patients after TIA or stroke, copeptin was a predictive factor of the recurrent vascular event [25]. Together, these data show that it may be beneficial to improve the ABCD2 score for TIA (age, blood pressure, clinical features, duration of TIA, and presence of diabetes) by adding copeptin values or monitoring the patients with large artery stenosis and high copeptin concentration [21,23]. 

In general, copeptin seems to be an effective biomarker in risk stratification after ischemic stroke and in predicting vascular events after TIA. However, copeptin is also associated with low specificity or low discrimination as a biomarker in other conditions. For instance, a few studies assessed the risk of stroke-associated infections using copeptin [20,170,171,172]. While higher levels of the biomarker were associated with a higher risk of complications, especially pneumonia, the results were unsatisfactory regarding diagnostic ability [20]. Copeptin alone had a similar predictive value observed with white blood count or C-reactive protein (CRP), and its addition to established scales resulted in a minor improvement in discrimination ability [170,171,172]. 

Likewise, applying copeptin as a diagnostic tool to distinguish stroke patients from stroke-free subjects has been tested with inconsistent results [19,22,173]. However, it may help to exclude vascular causes in patients with dizziness in the emergency department [173]. 

In summary, copeptin seems to be an effective biomarker opening an opportunity to improve stroke prognosis. It should be considered that the field of novel biomarkers regarding cerebrovascular disease management is growing rapidly. The combination of both circulating blood protein biomarkers and genetic testing can be used for cerebrovascular risk assessment and outcome stratification after an ischemic event [174,175,176]. Still, the interpretation of blood-based biomarkers should always be correlated with individual clinical judgment and the physician’s best knowledge to avoid the danger of undertreatment of patients who are believed to be at a high risk of a poor outcome [177]. 

## 6. Targeting Arginine Vasopressin Receptors in the Acute Phase of Ischemic Stroke

The mechanisms mentioned above, especially brain edema exacerbation, prompted the rationale for AVP receptor antagonism in stroke. The use of a selective V1aR antagonist (SR49059) in MCAO rodent stroke models improved neurological outcomes and reduced infarct area [30,135,178]. The histopathological findings, such as decreased brain water content in the infarct area, sodium shift into the brain, reduced AQP4 expression, and reduction of BBB disruption, could be a plausible explanation for the beneficial outcome of SR49059-treated animals. Notably, both histological (reduced brain water content and Na+ accumulation) and neurobehavioral (higher modified Garcia score and better performance in beam balance test and wire hanging test) outcomes were achieved when the V1aR antagonist was administered no more than one hour after the procedure [29,81,135,178]. The drug administration after three or six hours since the MCAO proved ineffective [30].

OPC-31260, a V2R antagonist, prevented water and sodium accumulation in the brain in both general cerebral hypoxia and SAH rat models. Moreover, in both studies, V2R antagonism enhanced plasma AVP increase in subjected rodents [27,28].

Tolvaptan is a clinically approved V2R antagonist, successfully used in hyponatremia treatment [34]. However, in the ischemic stroke mouse model, Tolvaptan and other selective V2R antagonists, apart from the established aquaretic effect, did not produce the neuroprotective effects displayed by V1aR antagonists [134,179,180]. 

Conivaptan is an FDA-approved V1aR and V2R antagonist [35]. The main indication for Conivaptan use is euvolemic hyponatremia in specific disorders, including SIADH [181]. In animal stroke models, Conivaptan improved neurological outcomes, reduced brain water content, and attenuated BBB disruption [179]. In a different study, AVP receptor antagonism reduced brain edema and BBB disruption and improved neurological deficits in mice subjected to MCAO. Importantly, in this study, Conivaptan proved effective when administered three hours after an infarct induction [182], suggesting it may also be effective when administered within a similar time window of standard stroke treatment (i.e., thrombolysis). Recently, Can et al. have shown evidence for Conivaptan superiority over mannitol in diuretic activity in ischemic stroke achieved by a 30-min common carotid occlusion. Moreover, Conivaptan decreased serum 2-Phospho-D-Glycerate-Hydrolase (NSE) and increased progranulin. The former is a clinical biomarker associated with post-injury brain dysfunction, whereas the latter is known for its neuroprotective properties [183]. Regarding the safety of Conivaptan in stroke, the standard dose of the drug (20 mg) administered every 12 h for 2 days in combination with standardized intracranial hemorrhage (ICH) management proved safe and well tolerated by the patients [184]. In a case report published by Hedna et al. presenting a patient with post-operational ICH, the addition of Conivaptan to ineffective conventional anti-edema therapy resulted in both conscious level and motor function improvement, as well as radiologically-assessed brain edema resolution with no reported side effects [185]. 

Overall, antagonism of just V1R or both V1R and V2R provides a better outcome in rodent stroke models than using V2R antagonists alone. Recent literature regards Conivaptan as a safe, mixed AVP receptor antagonist that has the potential to supplement stroke treatment. Nevertheless, phase II studies are needed to test the effectiveness of Conivaptan in stroke.

## 7. Discussion 

**Figure 1:**
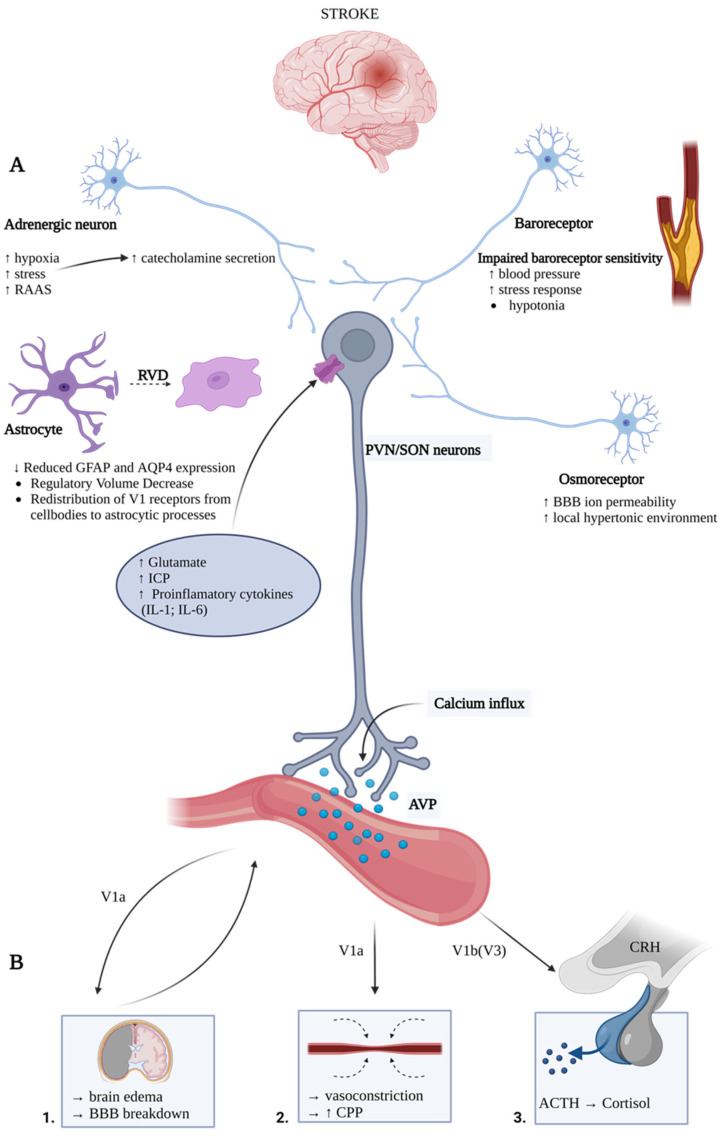
Main mechanisms causing arginine-vasopressin (AVP) release during stroke (**A**) and main local deteriorating effects caused by excessive AVP release (**B**). AVP neurons receive dense afferent input from baroreceptors, osmoreceptors, adrenergic, and glutaminergic neurons. Stroke induces the dysregulation of baroreceptors and the renin-angiotensin-aldosterone system, and increases the sympathetic stimulation of the paraventricular nucleus of hypothalamus (PVN) neurons (**A**). After the stroke, the initial disruption of the blood-brain barrier causes local hyperosmotic environment formation and stimulation of osmoreceptors (**A**). The release of AVP is also regulated by the interaction of astrocytes with AVP-containing neurons. During a stroke, the regulatory volume decrease of astrocytes occurs, followed by the downregulation of GFAP and AQP4 (**A**). Additionally, stroke causes the release of glutamate and pro-inflammatory cytokines, which stimulate AVP release (**A**). After the release, AVP activates the V1aR and V1b(V3)R located in the brain, brain vasculature, and pituitary, respectively (**B**). Upon release, AVP exacerbates brain edema formation (1). Consequently, the expanding brain edema can increase the intracranial pressure and exert direct pressure on adenohypophysis, further increasing the AVP release (1). Vasopressin causes time-dependent arteries and arterioles vasoconstriction and increases cerebral perfusion pressure (CPP) but not cerebral blood flow (CBF) (2). Vasopressin also acts synergistically with corticotropin (CRH), causing excessive cortisol release (3). Abbreviations: ACTH, adrenocorticotropic hormone; AQP4, aquaporin-4; AVP, arginine vasopressin; BBB, blood-brain barrier; CBF, cerebral blood flow; CPP, cerebral perfusion pressure; CRH, corticotropin-releasing hormone; GFAP, glial fibrillary acidic protein; IL-1, interleukin 1; IL -6, interleukin 6; PVN, paraventricular nucleus; RAAS, renin–angiotensin–aldosterone system; and RVD, regulatory volume decrease.

During the acute phase of stroke, AVP release is uncontrolled and larger than observed in physiological conditions. The mechanisms for AVP release in stroke are independent of plasma osmolality and involve a complex interplay and synergism between regulatory systems, systemic factors, and local components. Among them, AVP secretion underlying mechanisms include an interplay between the sympathetic and renin-angiotensin system; systemic changes in glycemia; and regional increase in glutamate excitability, as well as maladaptation of the neurovascular unit (reviewed in [11]). The resultant AVP hypersecretion is associated with many pathological detrimental phenomena, including (1) brain edema caused by hyponatremia aggravation, AQP4 expression modulation, and ion transporters’ function disruption, (2) BBB integrity disruption through MMP9 and VEGF upregulation (3) hypercortisolemia caused by HPA axis activation, (4) neuroinflammation through inflammatory cytokines upregulation and enhancement of inflammatory cells infiltration, and (5) vasoconstriction through direct V1a-mediated effect on cerebral blood vessels and the promotion of oxidative stress, causing cerebrovascular dysregulation (Figure 1, and Table 1 and Table 2). Using the stable peptide copeptin as a surrogate marker of AVP, studies have found an association between a higher concentration of copeptin in stroke patients (hence, AVP) with a higher risk of poor outcomes and all-cause mortality. Furthermore, patients with higher copeptin levels at the time of TIA were at a greater risk of developing a stroke or any cerebrovascular re-event. Altogether, the revised individual literature establishes the potential of using copeptin as a biomarker for AVP plasma levels.

Targeting the AVP receptor with pharmacological receptor antagonists, either individually or in combination, aims to prevent pathological events spurred by V1R and V2R activation. A mixed AVP antagonist, Conivaptan, achieved the best safety and neurological outcomes, providing the most significant degree of potential in stroke treatment. 

Of note, most of the beneficial effects of AVP receptor antagonists were achieved in ischemia-reperfusion models. Thus, it is reasonable to infer that AVP receptor antagonism-oriented therapy is more efficient when applied together with the recanalization of the occluded vessel. In line with this, we believe that future studies involving AVP receptor antagonists should focus on using AVP antagonists as an add-on therapy for standard recanalization (thrombolysis/thrombectomy) treatment. It is crucial to recognize the potential of the anti-edematous effects of AVP receptor antagonists in post-stroke secondary brain damage alleviation. We strongly feel that treatment of post-stroke brain edema using AVP receptor antagonists in concert with standard brain edema treatment could be a viable means of stroke outcome improvement and mortality rate reduction, especially in patients with refractory brain edema [185]. As demonstrated previously, a phase I clinical trial on Conivaptan use, along with standard treatment for reduction of perihematomal edema, has proven its safety and tolerability in patients treated with ICH. In our view, relatively large amounts of data pointing to the efficacy of AVP receptor antagonists in ischemic stroke animal models supports the rationale for future clinical trials in ischemic stroke patients.

**Table 2:** The main results of selective and non-selective vaptans administration in animal and human stroke studies.

Experimental Model/Type of Study	Antagonist (Dose)	Time of Administration	Results	Author
Mouse; 60 min tMCAO	Conivaptan (i.v 0.2 mg, 0.2 mg) and Tolvaptan (p.o 0.2 mg)	At reperfusion	▪ Both antagonists produced aquaresis; ▪ Conivaptan: ↑neurological deficits, ↓ BWC, ↓ BBB disruption	[179]
Mouse; 60 min tMCAO	V1R antagonist (i. cv. 500 ng, 1000 ng) V2R antagonist (i. cv. 1000 ng)	10 min after surgery	▪ V1R antagonist- ↓ infarct volume; ↓ BWC (1000 ng); ↑ AQP4 expression (1000 ng) ▪ V2R antagonist: no effect	[134]
Rat; 2 h tMCAO	SR49059 (6480, 720, 640 µL/h)	1 h before/after reperfusion	▪ ↓ BWC in infarct area (720, 640 µL/h); ↓ sodium shift; ↓ Potassium loss prevention (only in”before reperfusion” timing condition)	[29]
Mouse; ICH; collagenase injection model	SR49059 (0.5 mg/kg, 2 mg/kg)	1 h after surgery	▪ ↓ Cerebral edema at 24 h and 72 h post-ICH, ▪ ↑ Neurobehavioral function at 72 h post-ICH; ▪ ↓ BBB disruption at 72 h post-ICH; ↓ AQP4 expression	[135]
Mouse; 60 min MCAO	V1R antagonist (i.cv)V2R antagonist (i.cv)	5 min after MCAO	▪ V1R antagonist- ↓ infarct volume, ↓ brain edema formation, ↓ BBB disruption, ↓ functional deficits ▪ V2R antagonist: no effect	[180]
Rat; pMCAO	SR49059 (i.p. 30 mg/kg, 2 mg/kg)	Immediately, 1 h, 3 h, 6 h after MCAO	▪ ↓ Infarct volume, ↓ Neurological deficits, ↓ Brain edema (in immediate and 1 h time conditions) ▪ Higher dose (30 mg/kg) shows no advantage over 2 mg/kg (at 1 h after MCAO)	[30]
Rat; general cerebral hypoxia; bilateral common carotid ligation model	OPC-31260 (p.o 30 mg/kg)	Immediately after operation	▪ ↑ Survival rate; ↓ increase in BWC and accumulation of Na^+^; ↑ AVP level increase	[28]
Rat; SAH; autologous blood administration on cerebral cortex	OPC-31260 (p.o 30 mg/kg, 10 mg/kg)	Immediately after operation or every 8 h following operation	▪ ↓ Water and Na+ accumulation; ↑ AVP level in plasma increase	[27]
Rat; MCAO	5% HS bolus +5% HS (HS) Conivaptan (Con) Conivaptan + 5% HS (Con+HS) Conivaptan + 5% HS bolus+ 5% HS (Con+HSb)	6 h after MCAO—early group 24 h after MCAO—late group	▪ ↓ Infarct volume, ↓ Brain edema in HS and Con treated early groups and HS treated late group ▪ ↓ Microglia/macrophage activation in Con treated early group	[186]
Rat; 30 min tCCAO	Conivaptan (i.v. 10 mg/mL, 20 mg/mL)	At reperfusion	▪ Conivaptan has better diuretic activity than mannitol ▪ ↑ AVP level increase, ↓ serum NSE, ↑ serum PGRN	[183]
Mouse; 60 min tMCAO	Conivaptan (i.v. 0.2 mg/day)	3, 5, and 20 h after reperfusion	▪ ↓ Edema, ↓ Neurological deficits improvement, ↓ BBB disruption (when administered 3 h after reperfusion) ▪ No effect on edema/BBB disruption (when administered 5 and 20 h after reperfusion)	[182]
Phase I clinical study, ICH at risk of developing PHE	Conivaptan (20 mg) + standard management	Every 12 h for 2 days	▪ Conivaptan is safe to use with ICH standard treatment	[184]
Case report; refractory brain edema following hemorrhagic stroke	Conivaptan (i.v 20 mg in 30 min + 20 mg over 24 h)	2 days after hemorrhage detection	▪ Rapid clinical and radiological improvement	[185]

tMCAO, Transient middle artery occlusion; BWC, Brain water content; BBB, Blood-brain barrier; ICH, Intracranial hemorrhage; AQP4, Aquaporin-4; V1R, Vasopressin V1 receptor; V2R, Vasopressin V2 receptor; MCAO, Middle cerebral artery occlusion; pMCAO, Permanent middle cerebral artery occlusion; SAH, Subarachnoid hemorrhage; HS, Hypertonic saline; tMCAO, transient middle cerebral artery occlusion; PGRN, progranulin; NSE, Neuron-specific enolase; PHE, Perihematomal edema. Down arrows (↓) indicate a decrease of the respective results cited; up arrows (↑) indicate an increase.

A limited number of studies have focused on the possibility of using AVP receptor antagonists in animal models of hemorrhagic stroke. Although ischemic stroke is epidemiologically more frequent than hemorrhagic stroke, we suggest that the efficacy of vasopressin antagonists in the latter should also be investigated [187]. Thus, we encourage researchers to include hemorrhagic stroke models in their studies. 

In this review, we pointed out the involvement of AVP in some stroke-related pathophysiological events. The multiplicity of mechanisms involved in primary and secondary brain damage in stroke points out the still underexplored areas of possible AVP influence. We propose that further research should be undertaken in the following areas: (1) reperfusion injury, (2) neuroinflammation, (3) vasospasm, (4) oxidative stress, and (5) BBB disruption.

This review provides an update on understanding AVP involvement in ischemic stroke. Moreover, it highlights viable future research areas to explore. Our work would lend itself well to clinicians conducting clinical trials involving AVP receptor antagonists and researchers exploring the molecular mechanisms behind other neurological conditions. The ultimate goal of this review is to provide a springboard for further studies on the involvement of AVP in stroke settings and the implication thereof in modern medicine. In our view, the biggest challenge would be the time-consuming and cost-intensive process of clinical trials, particularly considering other competing research areas regarding stroke therapy, including neuroprotective drugs, safer/cheaper thrombolytic agents, and extended time windows protocols for thrombolysis/thrombectomy. Moreover, since the animal model for human ischemic stroke does not adequately reflect the human brain conditions in stroke, the discussed results of animal studies could be delusive, thus undermining the rationale for large-scale studies. To overcome these challenges, further experimental investigations are needed to establish the complete basis of the AVP mechanism of action in terms of stroke. For instance, the current animal stroke models should be improved by considering the age, metabolic state, and common comorbidities of stroke patients, as well as the anatomical and molecular differences between experimental animals and humans. Due to the significant burden of stroke in the world’s rapidly growing and aging population, the scientific fields studying novel stroke therapeutics and stroke pathomechanism are one of the central issues currently being researched. The line of research regarding the involvement of AVP in stroke is especially significant, given that some drugs that could putatively diminish the detrimental effect of AVP are widely available for treatment in other medical scenarios, thus making them easily accessible in case of a positive translation process. 

## 8. Conclusions

In conclusion, overlapping detrimental events occurring both in stroke and AVP hypersecretion models and the effectiveness of AVP receptor antagonists provide evidence to infer that AVP plays a vital role in stroke development. However, the indirect nature of the gathered data does not provide us with the essential details to determine the overall involvement of AVP-mediated mechanisms in stroke. Moreover, the differences between ischemic and hemorrhagic stroke regarding AVP release and the mechanism of action remain largely uncovered. Nevertheless, monitoring AVP levels and targeting AVP receptors are of paramount potential in clinical applications. Undoubtedly, molecular and prospective clinical studies are needed to discover the precise mechanisms behind AVP effects and integrate the measuring of copeptin or/and the use of AVP receptor antagonists in clinical practice.

Based on the data included in the sections of the manuscript, we can summarize that:

- A stroke is associated with the release of AVP into the blood in response to various endogenous stimuli;
- Overproduction of AVP is most likely detrimental to the brain, primarily due to the AVP-elicited brain edema;
- Copeptin is a surrogate marker of AVP and can be used as a prognostic biomarker of stroke outcome;
- AVP receptor antagonists can potentially be used as an add-on therapeutic intervention in stroke.

## Data Availability

Not applicable.
